# Dyslipidemia among patients with type 2 diabetes in Jordan: Prevalence, pattern, and associated factors

**DOI:** 10.3389/fpubh.2022.1002466

**Published:** 2022-11-08

**Authors:** Dana Hyassat, Saba Al-Saeksaek, Duha Naji, Awn Mahasneh, Yousef Khader, Mousa Abujbara, Mohammad El-Khateeb, Kamel Ajlouni

**Affiliations:** ^1^The National Center for Diabetes, Endocrinology, and Genetics, Amman, Jordan; ^2^Department of Public Health, Jordan University of Science and Technology (JUST), Irbid, Jordan

**Keywords:** dyslipidemia, diabetes, lipid profile, prevalence, associated factors

## Abstract

**Objectives:**

To determine the prevalence and patterns of dyslipidemia and its associated risk factors among patients with type 2 diabetes attending the National Center for Diabetes, Endocrinology, and Genetics (NCDEG).

**Methods:**

A cross-sectional study was conducted at the NCDEG in Amman, Jordan. A total of 971 patients with type 2 diabetes were included during the period September- December 2021. The socio-demographic data were collected through face-to-face interview questionnaire and anthropometric and clinical data were abstracted from medical records. The last three readings of lipid profile and HbA1C were abstracted from the medical records.

**Results:**

The overall prevalence of dyslipidemia among type 2 diabetic patients was 95.4%. The most common type of dyslipidemia was combined dyslipidemia (37.1%), with high triglycerides and low HDL-c (19.0%) being the most frequent type. Factors associated with hypercholesterolemia were diabetes duration ≤ 10 years, poor compliance to a statin, and HbA1c level (7–8%) (*P*-values: 0.008, 0.001, 0.021, respectively). Moreover, smoking and poor compliance with statin therapy were associated with high LDL-c level (*P*-values: 0.046 and 0.001, respectively). The presence of hypertension, high waist circumference, HbA1c level >8%, and diabetes duration ≤ 10 years were all associated with high triglyceride level (*P*-values: 0.008, 0.016, 0.011, and 0.018, respectively). Hypertension and HbA1c level >8% were associated with low HDL-c level (*P*-values: 0.010 and 0.011, respectively).

**Conclusion:**

The combination of high triglyceride and low HDL-c is the commonest lipid abnormality detected in patients with type 2 diabetes. An educational program that emphasizes the importance of adherence to a healthy lifestyle is strongly recommended. Further studies are needed to capture a wide range of factors that might influence dyslipidemia and glycemic control.

## Introduction

Type 2 diabetes is a global public health problem that is known to be associated with increased cardiovascular mortality and morbidity. People with diabetes have a 2 to 4-fold increase in the risk of ischemic heart disease, a 2-fold increase in stroke risk and a 4 to 8 years reduction in life expectancy (1, 2). Dyslipidemia, characterized by an abnormal lipid profile, is one of the major risk factors for cardiovascular disease in patients with diabetes (3), and is mainly due to increased free fatty acids flux secondary to insulin resistance (4). Metabolic syndrome with its associated insulin resistance leads to increased lipolysis by reducing inhibition of hormone-sensitive lipase in adipose tissue, thereby stimulating portal flux of free fatty acids to the liver (5, 6). These fatty acids in turn disrupt the activity of the hormone lipoprotein lipase causing an overproduction of triglyceride-rich lipoproteins [very-low-density lipoprotein (VLDL) and Chylomicrons] which are commonly associated with a reduction in HDL-c and an increase in small dense oxidized LDL-c levels (7, 8).

The prevalence of dyslipidemia among patients with type 2 DM is high (9). In a study conducted in Yemen, the prevalence of diabetic dyslipidemia among patients with type 2 DM was 85% and the common patterns of dyslipidemia in that study were high LDL-C followed by high serum cholesterol level (10).

The overall age-standardized prevalence of diabetes in Jordan increased from 13.0% in 1994 to 17.1% in 2004, 22.2% in 2009 and 23.7% in 2017 (11). In Jordan, one study in 2008 reported that over 90% of patients with type 2 diabetes had one or more types of dyslipidemia and the proportions of patients with type 2 diabetes who had hypercholesterolemia, low HDL-c, high LDL-c, and hypertriglyceridemia were 77.2, 83.9, 91.5, and 83.1%, respectively (12). In 2018, a national survey of 4,056 Jordanian adults reported that the prevalence rates of hypercholesterolemia, hypertriglyceridemia, high LDL-c, and low HDL-c were 44.3, 41.9, 75.9, and 59.5%, respectively (13). Hypercholesterolemia in Jordan almost doubled from 23% in 1994 to 44.3% in 2017, and hypertriglyceridemia increased from 23.8% in 1994 to 41.9% in 2017, indicating that dyslipidemia is widely prevalent among adult Jordanians and has substantially increased since 1994 (13).

There is a paucity of data available on the prevalence and patterns of dyslipidemia among patients with type 2 diabetes in Jordan. Therefore, this study was conducted to assess the prevalence and patterns of dyslipidemia and its associated factors among patients with type 2 diabetes in a country with a high and increasing prevalence of diabetes and a high rate of an unhealthy lifestyle.

## Methods

### Study design

A cross-sectional study was conducted at the National Center for Diabetes, Endocrinology, and Genetics (NCDEG), Amman-Jordan. All patients with type 2 diabetes (aged > 25 years) who attended the NCDEG during the period between September and December 2021 and had their last three readings of lipid profile documented in the medical records were included in the study. Patients with type 1 diabetes mellitus and women with gestational diabetes mellitus were excluded.

### Data collection

Data were collected using a structured questionnaire administered face-to-face. The questionnaire was adopted from WHO stepwise (STEPs) survey of non-communicable diseases and related literature (14). Patients' socio-demographic data including gender, age, marital status, occupation, and income were collected using face-to-face interviews. Anthropometric and clinical data were abstracted from medical records. These data included weight, height, body mass index, waist circumference at last visit, presence of other comorbidities such as hypertension, ischemic heart disease, and hypothyroidism, as well as diabetes complications (retinopathy, nephropathy, and neuropathy). Additional data including smoking history, alcohol consumption, type and duration of diabetes treatment, type as well as duration of anti-dyslipidemia treatment used (statin, fibrates, statin-fibrate combination, ezetimibe, and omega- 3 supplement), and compliance with statin treatment were collected using face to face interviews. The compliance was assessed by asking the patient one question “Do you consider yourself compliant to statin medication.” Those who answered “Yes” were considered compliant. The data collectors were trained in conducting a face-to-face interviews, abstraction of data from medical records, and ensuring data confidentiality.

### Laboratory investigations

The last three readings of full lipid profile, random blood sugar (RBS), glycated hemoglobin A1c (HbA1C), creatinine kinase (CK), and thyroid stimulating hormone (TSH) were abstracted from the medical records. Serum concentrations of total cholesterol, triglycerides, LDL-c, and HDL-c were retrieved by the automated spectrophotometer, COBAS INTEGRA, using commercial kits supplied by Roche Diagnostics.

### Variables definition

Anthropometric measurements, including height, weight, and waist circumference were measured while patients were wearing no shoes and light clothing. BMI was expressed as the quotient between weight (kg) and height squared (m^2^). Patients' BMI was classified according to WHO classification, as being normal (BMI; 18.5 to 24.9 kg/m^2^), overweight (BMI; 25 to 29.9 kg/m^2^), or obese (BMI ≥ 30 kg/m^2^) (15). Waist circumference was measured using a non-stretchable tape held in a horizontal plane around the abdomen at the level of the iliac crest at the end of a normal expiration. Waist circumference was considered high if it was higher than 94 cm in men and 80 cm in women (16). Hypertension was defined as systolic blood pressure > 130 mmHg, or diastolic blood pressure >80 mmHg, or if the patient was already taking anti-hypertensive medication (15).

Diabetes Mellitus (DM) was diagnosed if the patient had a fasting blood sugar >126 mg/dl (7 mmol/l) in two occasions or if the patient had a random plasma glucose >200 mg/dl (11.1 mmol/l) in the presence of classical symptoms of hyperglycemia, or had HbA1C > 6.5%. Furthermore, DM was considered to be controlled if the patient had HbA1C < 7.0% according to the American Diabetes Association (ADA) guidelines (15).

Dyslipidemia was defined according to the adult treatment panel III (ATPIII) as the presence of one or more of the following lipid abnormalities: Total cholesterol level ≥ 200 mg/dl, Triglyceride level ≥ 150 mg/dl, LDL-c levels ≥ 100 mg/dl, or HDL-c levels < 40 mg/dl or < 50 mg/dl in males and females, respectively, or if the patient was already on anti-dyslipidemic agents (17). Patients with dyslipidemia were further classified into isolated single-parameter dyslipidemia, combined-parameter dyslipidemia (two abnormal lipid parameters) and mixed-parameter dyslipidemia [elevations in LDL cholesterol and triglyceride (TG) levels combined with decreased levels of HDL cholesterol] (18).

The categorization of age and income was based on previous research conducted in patients with diabetes in Jordan. The diabetes duration and statin duration were categorized based on medians.

### Ethics

Ethical approval was obtained from the NCDEG research ethics committee. Informed consent was taken from all participants. Data were kept strictly confidential and were used only for scientific purposes.

### Statistical analysis

The data were analyzed using the Statistical Package for Social Sciences (SPSS, version 21). Mean and standard deviation were used to describe continuous variables and percentages were used to describe categorical variables. The association of different lipid abnormalities with different variables was determined using Chi-square (χ2) test. Multivariate logistic regression analysis was performed to assess the factors associated with different lipid abnormalities after adjusting for potential confounders. Variables were entered in the regression model using a forward stepwise regression approach that starts from the null model and adds a variable that improves the model the most one at a time. The criterion for predictor entry into the model was based on the F-statistic. Only significant variables at the alpha level of 0.05 were kept in the model. A *P*-value of < 0.05 was considered statistically significant.

## Results

### Participants' characteristics

This study included 971 patients with type 2 diabetes (46.5% males and 53.5% females) aged between 30 and 85 years [mean (SD): 58.4 (9.2) year]. Almost 41.4% of patients were older than 60 year. About 62.0% of participants had obesity, 27.4% were smokers while 90.9% had high waist circumference. The socio-demographic and clinical characteristics of the participants are presented in Table 1. Almost half of the patients (47.6%) were diagnosed with type 2 diabetes for more than 10 years. Around 85% of type 2 diabetes patients of this study were taking various types of lipid-lowering treatments (80.5% were on a statin, 10.7% on Fibrates, 9.5% on the statin-fibrate combination, 0.2% on Ezetimibe and 17.7% of them were taking omega-3 supplements).

**Table 1:** Socio-demographic and clinical characteristics of the study population (*N* = 971).

**Variable**	***N* (%)**
**Age (years)**	
<50	172 (17.7%)
50–60	397 (40.9%)
>60	402 (41.4%)
**Gender**	
Male	452 (46.5%)
Female	519 (53.5%)
**BMI (kg/m** ^2^ **)**	
Normal: <25	72 (7.4%)
Overweight: 25–29.9	297 (30.6%)
Obese: ≥30	602 (62.0%)
**Waist circumference (cm)**	
(M < 94, F < 80)	88 (9.1%)
(M ≥ 94, F ≥ 80)	883 (90.9%)
**Marital status**	
Married	826 (85.1%)
Single	32 (3.3%)
Divorced/ widow	113 (11.6%)
**Occupational status**	
Employed	236 (24.3%)
Not employed	735 (75.7%)
**Monthly income (JD)**	
< 300	294 (30.3%)
301–500	344 (35.4%)
>500	333 (34.3%)
**Diabetes duration (years)**	
>10	462 (47.6%)
≤ 10	509 (52.4%)
**Diabetes treatment**	
Oral Hypoglycemic agents (OHA)	501 (51.6)
Insulin only	30 (3.1%)
Insulin + OHA	440 (45.3%)
**Statin duration (years)**	
>5	420 (53.5%)
≤ 5	365(46.5%)
**Smoking**	
Yes	266 (27.4%)
No	705 (72.6 %)
**Level of education**	
High school or less	453 (46.7%)
More than high school	518 (53.3%)
**Received anti-dyslipidemia agent**	882 (84.7%)
Statins	782 (80.5%)
Fibrates	104 (10.7%)
Statin-fibrate combination	92 (9.5%)
Ezetimibe	2 (0.2%)
Omega-3	172 (17.7%)
**Comorbidities**	
Hypertension	662 (68.2%)
Ischemic heart disease	182 (18.7%)
Hypothyroidism	127 (13.1%)
At least one comorbidity	729 (75.1%)
**Diabetes complications**	
Retinopathy	234 (24.1%)
Neuropathy	266 (27.4%)
Nephropathy	147 (15.1%)
At least one complication	458 (47.2%)

### Prevalence of dyslipidemia

The prevalence of dyslipidemia (at least one abnormal lipid fraction) was 95.4%. The most common pattern of dyslipidemia was combined dyslipidemia (37.1% in total, 41.1% in males and 33.9% in females), followed by isolated dyslipidemia (27.8% in total, 26.0% in males and 29.5% in females) and mixed dyslipidemia (25.3% in total, 22.3% in males and 27.8% in females) (Table 2).

**Table 2:** Prevalence of different patterns of dyslipidemia among patients with type 2 diabetes, Jordan, 2021 (*N* = 789).

**Dyslipidemia**	**Male *N* (%)**	**Female *N* (%)**	**Total *N* (%)**
**Isolated dyslipidemia**	93 (26.0%)	127 (29.5%)	220 (27.8%)
High LDL-c	38 (10.6%)	57 (13.2%)	95 (12.0%)
Low HDL-c	39 (10.9%)	59 (13.7%)	98 (12.4%)
High TG	16 (4.5%)	11 (2.6%)	27 (3.4%)
**Combined dyslipidemia**	147 (41.1%)	146 (33.9%)	293 (37.1%)
High LDL-c + High TG	39 (10.9%)	43 (10.0%)	82 (10.4%)
High LDL-c + Low HDL-c	21 (5.9%)	40 (9.3%)	61 (7.7%)
High TG + Low HDL-c	87 (24.3%)	63 (14.6%)	150 (19.0%)
**Mixed dyslipidemia**
High LDL-c + Low HDL-c + High TG	80 (22.3%)	120 (27.8%)	200 (25.3%)
No dyslipidemia	38 (10.6%)	38 (8.8%)	76 (9.8%)
**Total**	358	431	789

LDL, Low-Density Lipoprotein; TG, triglycerides; HDL, High-Density Lipoprotein.

The most prevalent combined dyslipidemia was high Triglycerides plus low HDL-c (19% in total, 24.3% in males, and 14.6% in females), followed by high LDL-c plus high triglycerides (10.4% in total, 10.9% in males, and 10.0% in females), while the most common isolated dyslipidemia in the total population was low HDL-c, followed by high LDL-c (12.4 and 12.0%, respectively). Whereas, mixed dyslipidemia in the form of (high LDL-c plus low HDL-c plus high triglycerides) was found in 25.3% of the total population. Table 3 shows the prevalence of dyslipidemia according to studied characteristics.

**Table 3:** Prevalence of dyslipidemia according to selected variables among patients with type 2 diabetes, Jordan, 2021 (*N* = 971).

**Variable**	High total cholesterol^*^N (%)	High LDL^*^N (%)	High triglycerides^*^N (%)	Low HDL^*^N (%)
**Duration of diabetes (years)**				
>10	50 (13.2%)	215 (49.9%)	243 (56.4%)	251 (64.6%)
≤ 10	91 (23.3%)	310 (63.8%)	305 (62.5%)	260 (64.7%)
*P-*value	0.001	0.001	0.059	0.926
**HbA1c (%)**				
≤ 7	30 (13.5%)	144 (54.8%)	137 (52.5%)	132 (56.9%)
7.01–8	56 (21.1%)	188 (57.8%)	193 (59.4%)	153 (55.4%)
>8	55 (19.6%)	193 (58.7%)	218 (65.5%)	165 (58.1%)
*P*-value	0.078	0.611	0.006	0.816
**Smoking**				
Yes	39 (19.2%)	158 (62.2%)	165 (65.2 %)	136 (64.8%)
No	102 (18.1%)	367 (55.4%)	383 (57.5%)	375 (64.4%)
*P*-value	0.715	0.061	0.033	0.932
**Age (years)**				
< 50	31 (23.0%)	122 (74.4%)	98 (59.0%)	95 (68.3%)
50–60	63 (19.6%)	222 (58.7%)	226 (59.8%)	213 (64.7%)
>60	47 (15.1%)	181 (48.3%)	224 (59.7%)	203 (62.7%)
*P*-value	0.105	0.001	0.985	0.500
**Gender**				
Male	54 (15.3%)	219 (51.8%)	269 (63.9%)	228 (63.3%)
Female	87 (20.9%)	306 (61.9%)	279 (56.0%)	283 (65.5%)
*P*-value	0.047	0.002	0.015	0.524
**BMI (kg/m** ^2^ **)**				
>25	10 (18.5%)	38 (55.9%)	26 (37.7%)	26 (47.3%)
25–29.9	46 (19.2%)	165 (57.9%)	164 (57.5%)	137 (56.1%)
≥30	85 (17.9%)	322 (57.1%)	358 (63.4%)	348 (70.6)
*P*-value	0.907	0.948	0.001	0.001
**Hypertension**				
Yes	83 (15.6%)	330 (53.3%)	394 (63.3%)	371 (67.5%)
No	58 (24.6%)	195 (65.4%)	154 (51.9%)	140 (57.9%)
*P*-value	0.003	0.001	0.001	0.009
**Lipid-lowering drugs**				
No treatment	25 (23.6%)	109 (74.7%)	67 (44.7%)	53 (49.1%)
Statin only	75 (16.3%)	292 (54.0%)	292 (54.1%)	295 (62.1%)
Statin and fibrate only	12 (21.4%)	34 (56.7%)	56 (93.3%)	50 (86.2%)
Statin and omega	15 (16.3%)	52 (48.6%)	83 (78.3%)	67 (70.5%)
Fibrate only or fibrate and omega	4 (40.0%)	5 (45.5%)	11 (100.0%)	9 (81.8%)
Statin, fibrate, and omega	8 (32.0%)	15 (53.6%)	24 (85.7%)	22 (84.6%)
Omega only	2 (11.1%)	18 (75.0%)	15 (62.5%)	15 (78.9%)
P-value	0.097	0.001	0.001	0.001

* High total cholesterol (≥200) mg/dl, high LDL (Low Density Lipoprotein –cholesterol ≥ 100 mg/dl), high triglycerides (≥150) mg/dl, low HDL (High Density Lipoprotein–cholesterol: <40 mg/dl for males and <50 mg/dl for females).

### Factors associated with high cholesterol level ≥ 200 mg/dl

As shown in Table 4, factors associated with hypercholesterolemia (total cholesterol ≥ 200 mg/dl) were DM duration ≤ 10 years, poor compliance with statin treatment, and HbA1c level (7–8%). Patients with DM duration ≤ 10 years were 1.8 times more likely to have high cholesterol level as compared to those with DM duration >10 years (*P*-value = 0.008). Patients who were non-compliant with statin treatment were 2.8 times at more risk to have hypercholesterolemia as compared to those who were compliant with statin treatment (*P*-value = 0.001). Finally, patients with HbA1c (7–8 %) were ~2 times more likely to have a high cholesterol level (≥200 mg/dl) as compared with those with Hba1c level ≤ 7% (*P*-value = 0.021).

**Table 4:** Multivariate logistic regression analysis of the factors that are associated with high cholesterol, LDL and triglycerides, and low HDL.

	**Odds ratio**	**95% Confidence interval**	***P*-value**
**High total cholesterol**			
**DM duration (years)**			
>10	1		
≤ 10	1.8	1.12–3.0	0.008
**Compliant with taking statin medication**			
Yes	1		
No	2.8	1.8–4.6	0.001
**HbA1c (%)**			
≤ 7	1		
7.01–8	1.9	1.1–3.5	0.021
>8	1.5	0.8–2.8	0.154
**High LDL-cholesterol**			
**Age (years)**			
< 50	1		
50–60	0.5	0.3–0.9	0.035
>60	0.4	0.2–0.8	0.006
**Compliant with taking statin medication**			
Yes	1		
No	3.7	2.4–5.8	0.001
**Gender**			
Female	1		
Male	0.4	0.3–0.6	0.001
**Smoking**			
No	1		
Yes	1.5	1.008–2.2	0.046
**High triglycerides**			
**DM duration (years)**			
>10	1		
≤ 10	1.4	1.1–2.1	0.018
**Occupation**			
Not employed	1		
Employed	1.6	1.03–2.6	0.036
**Hypertension**			
No	1		
Yes	1.6	1.1–2.3	0.008
**Waist circumference (cm)**			
(M < 94), (F < 80)	1		
(M ≥ 94), (F ≥ 80)	1.9	1.1–3.4	0.016
**HbA1c (%)**			
≤ 7	1		
7.01–8	1.0	0.7–1.6	0.639
>8	1.6	1.1–2.5	0.011
**Low HDL-cholesterol**			
**Hypertension**			
No	1		
Yes	1.6	1.1–2.4	0.010
**HbA1c (%)**			
≤ 7	1		
7.01–8	1.1	0.8–1.8	0.450
>8	1.7	1.1–2.7	0.011

### Factors associated with high LDL level (≥ 100 mg/dl)

In the multivariate analysis (Table 4), poor compliance with statin treatment (OR = 3.7) and smoking (OR = 1.5) were associated with high LDL level. Older people aged between 50 and 60 year, and those above 60 year, were less likely to have high LDL levels, as compared to those under 50 year. Moreover, male gender was associated with lower odds of high LDL level as compared to the female gender.

### Factors associated with hypertriglyceridemia (triglyceride level ≥ 150 mg/dl)

DM duration ≤ 10 years (OR = 1.4), being employed (OR = 1.6), presence of hypertension (OR =1.6), high waist circumference (OR= 1.9) and HbA1C level >8% (OR = 1.6) were associated with increased odds of hypertriglyceridemia.

### Factors associated with low HDL level (male <40 mg/dl, female < 50 mg/dl)

The factors associated with low HDL level were the presence of hypertension and HbA1c level > 8%. Patients who had hypertension were 1.6 times more likely to have a low HDL level as compared to those with no hypertension. Poorly controlled diabetes was also associated with 1.7 times more odds of low HDL as compared to controlled HbA1c ≤ 7%.

## Discussion

This study showed that the vast majority of patients with type 2 diabetes had dyslipidemia (95.4%). The most common pattern of dyslipidemia in both males and females was combined dyslipidemia (high triglycerides and low HDL-c). Consistent with our finding, a study carried out in Pakistan (2016) among 200 patients (31.5% < 40 year and 68.5% ≥ 40 year) reported almost the same prevalence of dyslipidemia in hyperglycemic patients (95%) and that combined dyslipidemia with high triglycerides and low HDL-c was the most common type of dyslipidemia in males, while high LDL-c and low HDL-c were the most common in females (19). Previous studies (19–21) reported variable prevalence rates and patterns of dyslipidemia. This difference in the prevalence and patterns of dyslipidemia in patients with type 2 diabetes may be due to differences in the population studied, BMI, diabetes duration, genetic factors, socioeconomic development, and different definitions and cut-off values for lipids used.

The second most common pattern of dyslipidemia in both males and females was isolated dyslipidemia with low HDL-c (12.4%) followed by high LDL-c (12%). In agreement with our finding, a study conducted in India showed that the prevalence of low HDL-c was 71% followed by high LDL-c and hypertriglyceridemia (64, 47%, respectively) (22).

It has been reported in an Indian study that smoking is associated with an increase in oxidative modification of LDL, resulting in higher levels of oxidized atherogenic LDL and contributing for increasing the risk of atherosclerosis and coronary heart disease in smokers (23). In the present study, smokers were more likely to have high LDL levels as compared to non-smokers. Many studies found that low-density lipoprotein was significantly higher in smokers compared to non-smokers (24–26).

This study showed that patients ≥50 years were less likely to have LDL ≥100 mg/dl as compared to people aged <50 years. A decrease in LDL and total cholesterol levels with age was reported by many studies (27–29) and this might be explained by an age-related reduction in cholesterol absorption from the intestine or changes in dietary habits.

A higher prevalence of high LDL in women after middle age was observed in the present study, which was similar to previous other studies (30, 31). The Study of Women's Health Across the Nation (SWAN) also demonstrated an increase in LDL-c and total cholesterol within 1 year after the final menstrual period, which could lead to an increase in coronary or cardiovascular disease (32).

Dyslipidemia is one of the common presentation of poor glycemic control in patients with type 2 diabetes. The relative insulin deficiency or insulin resistance and the decrease in adiponectin level cause a decrease in the activity of lipoprotein lipase enzyme resulting in high levels of LDL, total cholesterol, triglycerides, and low levels of HDL. The presence of qualitative defects in LDL in form of high oxidized-atherogenic LDL, will also further amplify the risk of developing atherosclerosis and cardiovascular disease. One of the important findings in our study is the significant association between glycosylated hemoglobin (HbA1c), triglyceride and cholesterol levels. This finding was reported in other studies (33, 34).

Our study found a significant correlation between high blood pressure and high triglyceride levels. Consistent with this finding, a study in Bangladesh (2019) also found a positive correlation between serum triglyceride level and blood pressure (35). Moreover, the present study showed that individuals with high waist circumference have increased triglycerides level. In agreement with our study, a Korean study (2022) found a significant association between triglyceride level and waist circumference (36). Several mechanisms have been proposed to explain the association between hypertension, obesity and high triglyceride levels. Metabolic syndrome (MetS) forms a cluster of metabolic abnormalities including hypertension, atherogenic dyslipidemia, central obesity, and insulin resistance (37). Obesity and its associated insulin resistance and hyperinsulinemia are known to be associated with increased sympathetic activity (increased in circulating catecholamines release) which ultimately leads to an increase in plasma renin activity, that in turn increase the secretion of the powerful vasoconstrictor angiotensin II that is responsible for increasing peripheral vascular resistance and thus causes an increase in blood pressure (38). Additionally, hyperinsulinemia could directly increase renal tubular sodium reabsorption and stimulates aldosterone secretion leading to elevation in blood pressure. Angiotensin II could also act directly on the kidneys resulting in an increase in salt and water retention, an increase in blood volume and cardiac output, and the development of hypertension (39).

Moreover, hypertriglyceridemia also plays a key role in the development of oxidative stress that causes endothelial dysfunction which is associated with a reduction in nitric oxide production (the potent vasodilator) contributing to the development of high blood pressure (40, 41).

This study also demonstrated an association between dyslipidemia in form of (high total cholesterol and high triglycerides) and DM duration ≤ 10 years. On the contrary, a previous study performed in 2020 concluded that long DM duration was positively correlated with dyslipidemia (42). On the other hand, a study conducted in Northwestern Nigeria (2019) found that dyslipidemia status was not associated with age, sex, or duration of DM or hypertension (43).

Non-adherence to statin treatment is a widely recognized major public health concern, which could contribute to significant mortality, morbidity, and healthcare costs. Non-adherence to statin therapy was associated with higher risk to have hypercholesterolemia and high LDL level by our study. A study conducted in New Jersey that included 34,501 patients found that adherence to statin fell dramatically from 79% during the first 3 months to 56% after 6 months to 42% after 12 months (44). Moreover, a sub-analysis from a LEADD study in India showed sub-optimal adherence to ADA 2018 guidelines for the management of diabetic dyslipidemia. This calls for a more strict treatment approach to treating patients with dyslipidemia and diabetes using high-intensity statin to achieve more favorable outcomes (45).

## Study limitations and strengths

This study has multiple limitations that are inherited in the cross-sectional study. The association of factors with dyslipidemia should not be interpreted as causal because temporality cannot be established in a cross-sectional design. Another limitation is that the study was limited to patients in one center which might limit the generalizability of the study findings. Moreover, one should note that the compliance to statin therapy was self-reported by asking a single question. The main strength of this study is that the last three readings of full lipid profile, random blood sugar (RBS), glycated hemoglobin A1c (HbA1c), creatinine kinase (CK), and thyroid stimulating hormone (TSH) were abstracted from the medical records and analyzed.

## Conclusions

The most common type of dyslipidemia in patients with type 2 diabetes was combined dyslipidemia (high triglycerides and low HDL-c) followed by isolated dyslipidemia (low HDL-c). Smoking and poor compliance with statin therapy were associated with high LDL-c level. DM duration ≤ 10 years and high waist circumference were associated with high triglyceride level, while the presence of hypertension and uncontrolled DM were associated with both high triglyceride and low HDL-c levels.

## Recommendations

The high prevalence of obesity and overweight that was observed in our study suggests a need for an educational program that emphasizes the importance of adherence to a healthy lifestyle. Additionally, optimal care by routine monitoring of lipid profile and achieving good glycemic control should be helpful in controlling dyslipidemia among patients with type 2 diabetes. Further studies are needed to capture a wide range of factors that might impact dyslipidemia and glycemic control in patients with type 2 diabetes.

## Data availability statement

The original contributions presented in the study are included in the article/supplementary material, further inquiries can be directed to the corresponding author.

## Ethics statement

The studies involving human participants were reviewed and approved by the National Center (Institute) for Diabetes, Endocrinology and Genetics (NCDEG)/The University of Jordan. The patients/participants provided their written informed consent to participate in this study.

## Author contributions

DH wrote the manuscript, originator of the manuscript subject, and supervised the research. SA-S helped in developing the idea, setting the protocol, and supervised and edited the manuscript. DN collected the data. AM contributed to the conception and design of the study. YK performed the statistical analysis, approved the protocol from statistical point of view, analyzed the data, and approved the results. MA contributed in writing the manuscript, acquisition of data, and analysis and interpretation of data. ME-K contributed to the conception and design of the study and was responsible for lab analyses. KA originator of the manuscript subject and supervised the research. All authors contributed to the article and approved the submitted version.

## Conflict of interest

The authors declare that the research was conducted in the absence of any commercial or financial relationships that could be construed as a potential conflict of interest.

## Publisher's note

All claims expressed in this article are solely those of the authors and do not necessarily represent those of their affiliated organizations, or those of the publisher, the editors and the reviewers. Any product that may be evaluated in this article, or claim that may be made by its manufacturer, is not guaranteed or endorsed by the publisher.
